# Children with disabilities—An overview of their situation

**DOI:** 10.3402/qhw.v9.23899

**Published:** 2014-02-12

**Authors:** Ulrika Hallberg

**Affiliations:** Associate Professor Public Health, Sweden

Children with disabilities are a fast-growing group in all societies, and their situation is often strained not only by their disability itself but also by a lot of other challenging circumstances they have to face. What are included in these challenging situations and what can we as qualitative researchers do to improve their situation?

It is estimated that about 15–20% of all people in the world suffer from some kind of disability. That number is increasing rapidly, depending on relatively newly acquired knowledge in how to save extremely premature babies and how to save children with severe disabilities and/or diseases. Also, there is a general prolonged life expectation for persons who are severely sick and disabled and also new and better ways in diagnosing diseases and/or disabilities. Medically these children with disabilities often are fragile and hospitalized during repeated and/or long periods and are accordingly exposed to frequent medical treatments, which, in turn, are a risk for complications that can worsen their already challenged health. Caries and gingivitis are their most unmet health care needs, and these children still receive less and less advanced odontological treatments than others. These children also have a high risk of developing psychological ill-health, such as depression or anxiety, and may have difficulties in communicating these feelings to others.

Most of the children with disabilities come from economically challenged families, partly owing to their parent's limited abilities to work full-time. This is often depending on their strained life situation and partly owing to higher exposure for risk environments in poorer groups and thereby higher risks for diseases and/or disabilities in the children. Being a parent of a child with a disability constitutes a permanent stressor. This includes worries for the child's health and well-being, financial worries, marital problems, sleep disturbances, and worries for healthy siblings, which can lead to physical and/or psychological ill-health, especially in single mothers. Also, healthy siblings are at risk for psychological ill-health and socially challenging risk behavior owing to the strained life situation and increased responsibility level. Examples of this are frequent babysitting and limited possibilities for the parents in taking time for and acknowledging the siblings, and that is especially true for older sisters. Grandparents can however serve as moderators for the strained life situation in the family by listening to the parents, acknowledging the positive sides of the child with a disability, and by occasionally taking care of the healthy siblings. The child's school situation is often problematic; globally, these children's possibilities for attending school at all are limited and in the western societies the children are often included in schools that are not primary build for their needs. Also, the teachers are not primarily educated to meet these children's needs, which can lead to educational underachievement in the children. Further, children with disabilities are often excluded from play and other kind of interactions with peers without disabilities and can even be teased or bullied, which can lead to a decreased self-esteem. In addition, children with disabilities suffer an increased risk of maltreatment and abuse owing to challenging behavior and strained caregivers.

There is an enormous amount of research conducted in this area which have led to increased knowledge and understanding for these children's situation, including their families’ situations. This enormous amount of research is also part of the reason why most of these children lead better lives today than they did historically. However, still we are far away from the situation where children with disabilities and their families have the same possibilities as others. One way to get there is conducting more research that is up to date and of high quality. Examples of areas that are especially missing are deeper knowledge concerning psychological ill-health in these children, the situation of single parents and older siblings along with the school situation. All the mentioned areas seem to be appropriate to study by using different qualitative methods and here can we as qualitative researchers hopefully continue the work to make a difference for these children and their families also in the future.

*Ulrika Hallberg* Associate Professor Public Health, Sweden

